# NADPH oxidase 2 is necessary for chronic intermittent hypoxia‐induced sternohyoid muscle weakness in adult male mice

**DOI:** 10.1113/EP090536

**Published:** 2022-07-11

**Authors:** Sarah E. Drummond, David P. Burns, Sarah El Maghrani, Oscar Ziegler, Vincent Healy, Ken D. O'Halloran

**Affiliations:** ^1^ Department of Physiology School of Medicine College of Medicine & Health University College Cork Cork Ireland

**Keywords:** chronic intermittent hypoxia, NADPH oxidase, sternohyoid, upper airway

## Abstract

**New Findings:**

- **What is the central question of this study?**
  Exposure to chronic intermittent hypoxia (CIH) evokes redox changes, culminating in impaired upper airway muscle function: what is the specific source of CIH‐induced reactive oxygen species?
- **What is the main finding and its importance?**
  Profound sternohyoid muscle dysfunction following exposure to CIH was entirely prevented by apocynin co‐treatment or NADPH oxidase 2 (NOX2) deletion. The results have implications for human obstructive sleep apnoea syndrome and point to antioxidant intervention, potentially targeting NOX2 blockade, as a therapeutic strategy.

**Abstract:**

Exposure to chronic intermittent hypoxia (CIH) evokes redox changes, culminating in impaired upper airway muscle function. We sought to determine if NADPH oxidase 2 (NOX2)‐derived reactive oxygen species underpin CIH‐induced maladaptive changes in upper airway (sternohyoid) muscle performance. Adult male mice (C57BL/6J) were assigned to one of three groups: normoxic controls (sham); CIH‐exposed (CIH, 12 cycles/hour, 8 h/day for 14 days); and CIH + apocynin (NOX2 inhibitor, 2 mM) given in the drinking water throughout exposure to CIH. In addition, we studied sham and CIH‐exposed NOX2‐null mice (B6.129S‐*CybbTM^1Din^* ^/J^). Profound sternohyoid muscle dysfunction following exposure to CIH was entirely prevented by apocynin co‐treatment or NOX2 deletion. Exposure to CIH increased sternohyoid muscle NOX enzyme activity, with no alteration to the gene or protein expression of NOX subunits. There was no evidence of overt oxidative stress, muscle regeneration, inflammation or atrophy following exposure to CIH. We suggest that NOX‐dependent CIH‐induced upper airway muscle weakness increases vulnerability to upper airway obstruction. Our results have implications for human obstructive sleep apnoea syndrome and point to antioxidant intervention, potentially targeting NOX2 blockade, as a therapeutic strategy.

## 1 INTRODUCTION

Obstructive sleep apnoea syndrome (OSAS) is the most prevalent form of sleep disordered breathing (White & Younes, 2012). Repetitive upper airway collapse throughout the night cycle results in recurrent oxygen desaturations, with the frequency of events defining mild, moderate and severe sleep apnoea (Veasey & Rosen, 2019). Chronic intermittent hypoxia (CIH), resulting from repetitive airway collapse, is widely considered to be the driving force behind the detrimental morbidities observed in patients with OSAS. Exposure to CIH mediates multi‐system dysfunction associated with OSAS, elevating its status to that of a major public health risk (Lavie, 2009).

Reactive oxygen species (ROS), produced following repeated cycles of hypoxia and re‐oxygenation, are mediators of oxidative stress. Indeed, OSAS is considered an oxidative stress disorder (Lavie, 2003). Exposure to CIH drives a pro‐oxidant state resulting in aberrant plasticity at multiple levels of the respiratory control system (O'Halloran, 2016). Upper airway dilator muscles are pivotal in the maintenance of airway patency. Impaired function of these striated muscles of breathing contributes to perpetuated respiratory pathology in OSAS (Bradford et al., 2005; Petrof et al., 1996). The structure and function of upper airway muscles is altered in patients with OSAS, with these functional deficits subsequently contributing to upper airway collapse (Carrera et al., 1999; Sériès et al., 1996). Several studies have demonstrated CIH‐induced muscle weakness and/or fatigue in representative upper airway muscles including the geniohyoid, genioglossus and sternohyoid (Ding & Liu, 2011; Dunleavy et al., 2008; Jia & Liu, 2010; Liu et al., 2005; Liu et al., 2009; McDonald et al., 2015; Pae et al., 2005; Skelly et al., 2012b; Wang et al., 2013; Zhou & Liu, 2013). Antioxidant supplementation ameliorates or prevents upper airway muscle dysfunction following exposure to CIH in rat models (Dunleavy et al., 2008; Liu et al., 2005; Skelly et al., 2012b, 2013).

There is a paucity of information pertaining to the specific molecular mechanisms underlying CIH‐induced ROS‐dependent upper airway muscle weakness. ROS have an important homeostatic role in the generation of skeletal muscle force. ROS are produced by a variety of cellular complexes including mitochondria, xanthine oxidase, phospholipase A2 and NADPH oxidase (NOX) (Di Meo et al., 2016). The NOX family of enzymes is composed of NOX1–5 and DUOX1 and 2, which have explicit roles in ROS production. NOX2 is the most widely expressed NOX isoform throughout the body. This extends to skeletal muscle with NOX2 subunit expression demonstrated in the sarcoplasm and t‐tubules, highlighting the potential role of NOX2‐derived ROS in alterations to excitation–contraction coupling mechanisms (Hidalgo et al., 2006; Xia et al., 2003). Hypoxia is a potent activator of NOX2 and exposure to CIH has been shown to increase NOX2 and p47phox expression in the sternohyoid muscle of rats (Williams et al., 2015). Interestingly, NOX subunits are upregulated in the diaphragm of patients with chronic heart failure associated with protein oxidation (Ahn et al., 2017) and p47phox deletion prevents diaphragm muscle weakness in mice with heart failure (Ahn et al., 2015). Moreover, CIH‐induced NOX2‐dependent maladies have been demonstrated in various tissues including the brain, heart and testes (Hayashi et al., 2008; Zhan et al., 2005; Zhang et al., 2013).

In the current study, we exposed adult male mice to a well‐established, mild‐to‐moderate paradigm of CIH. We employed pharmacological and transgenic approaches to determine if NOX2‐derived ROS underpin CIH‐induced impairments in contractile function of the sternohyoid (representative upper airway muscle). We hypothesised that exposure to CIH results in upper airway muscle dysfunction and that blockade or deletion of NOX2 prevents CIH‐induced maladaptive changes to muscle contractile function. We further hypothesised that exposure to CIH would cause redox‐dependent transcriptional changes in genes essential to muscle function.

## 2 METHODS

### 2.1 Ethical approval

Procedures on live animals were performed under licence from the Government of Ireland Department of Health (B100/4498) in accordance with National and European legislation (2010/63/EU) following approval by University College Cork Animal Research Ethics Committee (AEEC no. 2013/035).

### 2.2 Chronic intermittent hypoxia animal model

C57BL/6J male mice (9 weeks old) were purchased from Envigo (Bicester, UK), and assigned to one of three groups: normoxic control (sham; *n* = 20), CIH‐exposed (CIH; *n* = 20) and CIH + apocynin (CIH + APO; *n* = 20). Animals were conventionally housed in temperature‐ and humidity‐controlled rooms, operating on a 12 h light–12 h dark cycle with food and water available ad libitum. For daily gas treatment, mice were housed in standard cages placed in commercially designed hypoxia chambers (Oxycyler, Biospherix, Lacona, NY, USA). Exposure to CIH consisted of the cycling of gas in environmental chambers from normoxia (21% O_2_) for 210 s to hypoxia (5% O_2_ at the nadir) over 90 s (12 cycles/h) for 8 h/day during light hours for 14 consecutive days as previously described (Lucking et al., 2014). CIH + APO group also received the NOX2 inhibitor apocynin in the drinking water (2 mM) for the duration of the exposure to CIH. The drug solution was made up fresh and changed daily. The sham group was exposed to 21% O_2_ in parallel. In separate studies, 9‐week‐old NOX2‐null male mice (B6.129S‐Cybb^tm1Din^/J backcrossed on C57 for multiple generations) were purchased from The Jackson Laboratory (Bar Harbor, ME, USA) and assigned to a sham (NOX2 knockout (KO) sham; *n* = 12) or CIH (NOX2 KO CIH; *n* = 12) exposure. Due to the immunocompromised nature of NOX2 deficient mice, these mice were housed in individually ventilated cages when not undergoing exposure to CIH.

Exposure to normoxia or CIH began at 11 weeks of age and all mice were studied the day after 14 consecutive days of exposure. Mice were weighed daily to monitor for any substantial deleterious effects of exposure to CIH on body mass. On ‘day 15’, breathing was assessed by whole body plethysmography on a subset of mice, as described elsewhere (Drummond et al., 2021). Thereafter, mice were anaesthetised with 5% isoflurane in air and killed humanely by cervical spinal dislocation. Sternohyoid (representative upper airway dilator muscle) muscles were excised and used for *ex vivo* muscle function analysis (Section 2.3) or were snap frozen in liquid nitrogen and stored at −80°C for further tissue processing prior to gene (Section 2.4) and protein (Sections 2.5, 2.6 and 2.7) analysis.

### 2.3 *Ex vivo* muscle function analysis

#### 2.3.1 Muscle dissection and preparation

To excise sternohyoid muscles, a ventral incision was made from the genu of the mandible to the sternum. Closed forceps were delicately placed under the paired sternohyoid muscles to gently tease them away from the trachea. The muscles were cut at the rostral and caudal boundaries and immediately placed in a storage bath of hyperoxic (95% O_2_–5% CO_2_) Krebs solution (NaCl 120 mM, KCl 5 mM, calcium gluconate 2.5 mM, MgSO_4_ 1.2 mM, NaH_2_PO_4_ 1.2 mM, NaHCO_3_ 25 mM, glucose 11.5 mM and d‐tubocurarine 25 μM) at room temperature. The paired sternohyoid muscles were separated longitudinally along the natural midline, separating the two muscles (∼2 mm each). Muscles were returned to the hyperoxic Krebs solution for 10–15 min to recover from dissection. Muscles were suspended vertically between two platinum plate electrodes in a water‐jacketed tissue bath at 35°C containing Krebs solution and were continuously aerated with hyperoxia (95% O_2_–5% CO_2_). Using non‐elastic string, one sternohyoid muscle was attached to a lever connected to a dual‐mode force transducer (Aurora Scientific Inc., Aurora, ON, Canada). Muscles were allowed an equilibration time of 15 min in the muscle bath before the experimental protocol was initiated.

#### 2.3.2 Isometric protocol

Muscle function was examined by employing a range of protocols previously described (Burns et al., 2018, 2017, 2019). Sternohyoid muscles from all experimental groups (sham, CIH, CIH + APO, NOX2 KO sham and NOX2 KO CIH; *n* = 8–10 per group) were examined. When assessing isometric muscle contractions, the force transducer remained at maximum tension (>100% load). Muscle strips were stimulated at supramaximal voltage via the platinum plate electrodes. The optimal length (*L*_o_), the length which produces the maximum twitch force, was obtained by stimulating the muscle supra‐maximally for 1 ms. The length of the muscle was adjusted using a micro‐positioner between each of these stimulations and once the *L*_o_ was achieved, the muscle remained at this length for the duration of the protocol. A single twitch stimulation was applied from which twitch force and contractile kinetics including contraction time (CT; time to peak force) and half‐relaxation time (½RT; time for force to decay by 50%) were determined. Following this, an isometric tetanic contraction was evoked by supramaximal stimulation at a frequency of 100 Hz for 300 ms to determine the peak tetanic force (*F*_max_). Specific force was calculated in N/cm^2^ of estimated muscle cross sectional area (CSA). The CSA of each strip was determined by dividing the muscle mass (weight in grams) by the product of muscle *L*_o_ (cm) and muscle density (assumed to be 1.06 g/cm^3^).

#### 2.3.3 Isotonic protocol

Throughout the isotonic protocol, the force transducer was set to varying degrees of tension (0–100% load). Initially, the force transducer was set to its minimum tension (0%) and the muscle was stimulated to contract against a load that equated to 0% of its *F*_max_. This load was increased in a step‐like manner (5%, 10%, 20%, 30%, 40%, 60%, 80%, 100%) % of *F*_max_. A contraction was elicited at each step and 30 s was allowed between each contraction to allow the muscle to fully return to *L*_o_. The length of shortening was defined as the maximum distance of shortening during the contraction and shortening velocity was derived from the distance of shortening during the first 30 ms of shortening during a contraction (when velocity is maximal). Peak shortening length and velocity were achieved at 0% of *F*_max_. Peak specific shortening (*S*_max_) was defined as the length of shortening per optimal length (*L*/*L*_o_). Peak specific shortening velocity (*V*_max_) was defined as *L*_o_ per second. From this protocol, we calculated the work (force × shortening) and power‐generating capacity (force × shortening velocity) of the sternohyoid muscle at each step. From the work‐load and power‐load relationships achieved through this protocol, peak work and peak power were extrapolated. Peak specific mechanical work (*W*_max_) was calculated as joules/cm^2^ and peak specific mechanical power (*P*_max_) as watts/cm^2^.

### 2.4 Quantitative reverse transcription–PCR

#### 2.4.1 RNA extraction and preparation

Sternohyoid muscles from all experimental groups (sham, CIH, CIH + APO, NOX2 KO sham and NOX2 KO CIH; *n* = 6–9 per group) were examined. Samples were removed from storage at −80**°**C and immediately weighed. Samples ranging from 20 to 40 mg were homogenised on ice in Tripure Isolating Reagent (Roche Diagnostics Ltd, Burgess Hill, UK) using a general laboratory homogeniser (Omni‐Inc., Kennesaw, GA, USA). Homogenates remained on ice for 20 min with intermittent vortexing to promote cell lysis. Total RNA was isolated from homogenates using the phenol–chloroform method in accordance with the manufacturer's instructions. An additional chloroform wash step was performed during phase separation. The quantity (ng/μl) and purity of isolated RNA (260:280 and 260:230 ratios) were assessed by spectrophotometry using a Nanodrop 1000 (Thermo Fisher Scientific, Waltham, MA, USA). The integrity of isolated RNA was assessed by visualisation of distinct 18S and 28S ribosomal RNA bands using an agarose gel electrophoresis system (E‐gel, Thermo Fisher Scientific).

#### 2.4.2 cDNA synthesis

Sternohyoid muscle RNA was reverse transcribed to cDNA using a Transcriptor First Strand cDNA Synthesis Kit (Roche Diagnostics) in accordance with the manufacturer's instructions.

#### 2.4.3 Quantitative reverse transcription–PCR

cDNA was amplified using Realtime ready Catalog or Custom assays (Roche Diagnostics) shown in Table 1 and Fast Start Essential Probe Master (Roche Diagnostics) as per the manufacturer's instructions. Briefly, all reactions were carried out in duplicate on a 96‐well plate and consisted of 5 μl cDNA and 15 μl master mix using the Lightcycler 96 (Roche Diagnostics). RNA negatives, reverse transcription negatives, cDNA negatives (no template controls) and plate calibrators were used on every plate. Quantification cycle values (*C*_q_) obtained from experiments for the genes of interest (Table 1) were normalised to that of a reference gene, *Hprt1*, to account for variations in input amounts of RNA/cDNA and the efficiency of reverse transcription. *Hprt1* was found to be the most stable gene of the candidate reference genes screened in consideration of gas exposure (hypoxia), drug treatment (apocynin) and genotype (NOX2 null). The relative gene expression was calculated using the ∆∆*C*_T_ method (normalised expression of the gene of interest to that of the reference gene), with a change in expression shown as a fold change relative to the control group (sham exposure).

**TABLE 1:** Assay details for genes of interest

Gene name	Gene symbol	Assay ID
NOX enzymes		
NOX1	*NOX1*	310986
NOX2	*Cybb*	317885
NOX4	*NOX4*	300795
p22phox	*Cyba*	317890
p47phox	*Ncf1*	301105
p67phox	*Ncf2*	317897
p40phox	*Ncf4*	317894
Rac	*Racgap1*	310907
Duox1	*Duox1*	317891
Duox2	*Duox2*	317888
Atrophy		
Atrogin‐1	*Fbxo32*	317844
MuRF‐1	*Trim63*	317843
Autophagy		
BNIP3	*Bnip3*	311465
LC3B	*Map1lc3b*	317920
GABARAPL1	*Gabarapl1*	317923
Mitophagy		
PINK‐1	*Pink1*	331846
PARK‐2	*Park2*	317264
Inflammation		
NFκB	*Nfkb1*	300085
Antioxidant		
SOD1	*Sod1*	310738
SOD2	*Sod2*	310295
Catalase	*Cat*	310718
Nrf2	*Nfe2l2*	313377
Muscle differentiation		
Myogenin	*Myog*	313501
Myostatin	*Mstn*	318626
MyoD	*Myod1*	313570
MEF2C	*Mef2c*	318629
IGF1	*Igf1*	313359
Sirtuin‐1	*Sirt1*	310480
Reference		
HPRT1	*Hprt1*	307879

Real‐time ready catalogue and custom assays from Roche Diagnostics used for cDNA amplification.

### 2.5 Western blotting

#### 2.5.1 Protein extraction and quantification

Sternohyoid muscles were weighed and homogenised with 8 × 10 s bursts using a general laboratory homogeniser (Omni‐Inc., Kennesaw, GA, USA) in modified ice cold radioimmunoprecipitation assay (RIPA) buffer containing: RIPA (25 mM Tris–HCl pH 7.6, 150 mM sodium chloride, 1% NP‐40, 1% sodium deoxycholate, 0.1% SDS), deionised water, protease inhibitor cocktail (104 mM 4‐benzenesulfonyl fluoride hydrochloride (AEBSF), 80 μM aprotinin, 4 mM bestatin, 1.4 mM E‐64, 2 mM leupeptin, 1.5 mM pepstatin A), phosphatase inhibitor cocktail (200 mM sodium fluoride, 5 mM sodium orthovanadate) and 100 mM phenylmethylsulfonyl fluoride (Sigma Aldrich, Arklow, Wicklow, Ireland) using a 10% w/v ratio. Homogenates were allowed 20 min lyse time on ice with intermittent vortexing at 4‐min intervals. Samples were centrifuged (U‐320R centrifuge Boeckel + Co, Hamburg, Germany) for 20 min at 4°C at 15,366 *g* and the supernatant was stored −80°C. The protein concentration of each sample was determined using a bicinchoninic acid (BCA) protein quantification assay (Thermo Fisher Scientific), as per the manufacturer's instructions. Absorbance was measured at 562 nm using a SpectraMax‐M3 spectrophotometer (Molecular Devices, Sunnyvale, CA, USA).

#### 2.5.2 Gel electrophoresis

Western blot analysis was carried out on sternohyoid muscle homogenates from CIH‐exposed and sham mice (*n* = 5–7 per group). Samples were diluted based on their protein concentration and mixed with an equal volume of 2× SDS‐PAGE loading buffer (4% SDS, 200 mM dithiothreitol, 20% glycerol, 100 mM Tris‐Cl (pH 6.8), 0.2% Bromophenol blue) and boiled at 100°C for 5 min on a dry heating block (Techne, London, UK). A total of 15 μg of protein from each sample was resolved on 10% SDS‐polyacrylamide gels (deionised water, 30% acrylamide, 1.5 M Tris pH 8.8, 10% SDS, 10% ammonium persulfate). Resolved proteins were then electrophoretically transferred onto nitrocellulose membranes (Bio‐Rad). Membranes were incubated in 0.1% (w/v) Ponceau S in 5% acetic acid to reversibly stain the transferred proteins to assess equal protein loading and transfer and were digitally photographed for densitometric analysis. The membranes were blocked for 1 h in TBST (20 mM Tris–HCl, pH 7.6, 150 mM NaCl, 0.1% Tween‐20) containing 5% non‐fat dried milk, and were incubated overnight with the primary antibody specific for the protein of interest as follows: anti‐NOX2, anti‐NOX4; 1:10000, 1:2000 (Abcam, Cambridge, UK) in 5% BSA/5% non‐fat dried milk. The next day, membranes were incubated for 1 h at room temperature with a 1:2000 dilution of horseradish peroxidase‐linked anti‐mouse secondary antibody (Cell Signaling Technology, Danvers, MA, USA) in 5% non‐fat dried milk/TBST. Bands were visualised using enhanced chemiluminescence (ECL Plus, GE Healthcare, Buckinghamshire, UK) and exposure to chemiluminescence‐sensitive film (Kodak, Rochester, New York, USA). Films were developed, digitally photographed and densitometric analysis of bands of interest was performed (QuantityOne, Bio‐Rad). Band intensities of proteins of interest were normalised to the intensities of the corresponding Ponceau S staining proteins, which were also measured by densitometric analysis to adjust for protein loading, allowing comparative analysis between sham and CIH‐exposed muscles. Results are expressed as optical density (OD)/Ponceau S (arbitrary units; a.u).

### 2.6 Spectrophotometric assays

#### 2.6.1 Protein extraction and quantification

Sternohyoid muscles were homogenised, and protein was quantified using the protocol previously described in Section 2.5.1.

#### 2.6.2 NADPH oxidase activity

NOX enzyme activity was assessed in sternohyoid muscle homogenates from sham and CIH‐exposed mice (*n* = 8 per group) using a cocktail mixture containing: nitroblue tetrazolium (NTB, 2.2 mM in water), Tris–HCl pH8 (2.8 mM) and diethylene‐triamine‐penta‐acetic acid (1.3 mM in Tris–HCl) and a fresh solution of NADPH (1 mM). A total of 20 μl of muscle homogenate, 250 μl of cocktail mixture and 30 μl of NADPH were added per well in a black 96‐well plate. The plate was gently shaken at room temperature for 2 min following which absorbance was measured in a SpectraMax‐M3 spectrophotometer at 560 nm at 1‐min intervals for 30 min. NOX activity corresponded to the slope of the formation of formazan blue over time. Data were expressed as enzymatic activity per mg of protein in the sample, previously determined by BCA assay, per minute.

#### 2.6.3 Citrate synthase activity

Citrate synthase is an enzyme that catalyses the reaction between acetyl coenzyme A (acetyl CoA) and oxaloacetic acid to produce citric acid. It is the initial enzyme of the tricarboxylic acid (TCA) cycle and is commonly used as a marker of mitochondrial integrity. Citrate synthase activity was examined in sternohyoid muscle homogenates from sham and CIH‐exposed mice (*n* = 8 per group). The experimental procedure for the citrate synthase activity assay was performed in accordance with the manufacturer's instructions (CS0720; Sigma‐Aldrich). Data were expressed as enzymatic activity per μmole per min per mg protein in the sample, previously determined by BCA assay.

#### 2.6.4 Thiobarbituric acid reactive substances

Thiobarbituric acid reactive substances (TBARS) are degradation products of fats which are routinely used as a marker of lipid peroxidation, an indirect measurement of oxidative stress. Levels of TBARS were examined in sternohyoid muscle homogenates from sham and CIH‐exposed mice (*n* = 8 per group). Malondialdehyde (MDA) of a known concentration was used to generate a standard curve. Next, 50 μl of thiobarbituric acid (TBA, 50 mM) was added to 50 μl of muscle homogenate and the solution was incubated for 60 min at 97°C on a dry heating block. Samples were subsequently cooled on ice, and 75 μl of methanol: 1 mM NaOH (91: 9) was added to the solution. Samples were then centrifuged at 704 *g* and 70 μl of the resultant supernatant was added per well in a black 96‐well plate. The plate was read in a SpectraMax‐M3 spectrophotometer using 523/553 excitation/emission settings and data were compared with standards. Data are expressed as nM TBARS per mg of protein in the sample, previously determined by BCA assay.

### 2.7 Cell signalling assays

#### 2.7.1 Protein extraction and quantification

Sternohyoid muscles were homogenised and prepared using the same protocol described in Section 2.5.1 with some minor optimisation alterations. These included using a 2.5% w/v ratio of tissue to RIPA buffer, using twice the amount of phosphatase inhibitor and a 1:3 dilution of homogenates for the BCA assay.

#### 2.7.2 Hypertrophy, atrophy and hypoxia‐inducible factor signalling assays

Cell signalling assays were performed using a phospho‐forkhead box‐3a (FOXO‐3a); a mitogen‐activated protein kinase phosphoprotein panel–phospho‐p‐38, phospho‐extracellular signal‐regulated kinases 1 and 2 (ERK1/2), and phospho‐c‐Jun N‐terminal kinase (JNK); and total hypoxia‐inducible factor‐1α (HIF‐1α) assay (Meso Scale Diagnostics, Rockville, MD, USA). The assays quantify the total protein or phosphoprotein content of the signalling proteins listed above. These assays were carried out on sternohyoid muscle homogenates (*n* = 8 per group) from mice exposed to 2 weeks of CIH or normoxia (sham). The Mesoscale multiplex format assay (Meso Scale Diagnostics) allows the measurement of all three of these phospho‐proteins in one well. A host of capture antibodies against various proteins of interest are pre‐coated on distinct spots in each well of a 96‐well plate. Singleplex versions of the assay described above function by the same principle, measuring only one target. Briefly, all wells of the plate were initially blocked for 1 h and subsequently washed out three times. A total of 25 μg of sample was added per well in duplicate and the plate was sealed and incubated with vigorous shaking (7–78 *g*) at room temperature for 3 h. This enabled the analyte to be captured by the immoblilised capture antibodies on the surface of the electrodes on the bottom of the well. Following this, the wells were washed out again and an antibody detection solution containing antibodies for each protein of interest was added. These detection antibodies are bound to an electrochemiluminescent compound that acts as a tag. The detection antibody binds to the protein of interest which is already bound to the capture antibody on the surface of the well, thus creating a sandwich immunoassay. Following a 1‐h incubation the wells were washed again and read buffer was added to provide the correct chemical environment for electrochemiluminescence to occur. The plate was loaded into the MSD Sector Imager where a voltage is rapidly applied to the plate's electrodes resulting in the labels attached to these electrodes emitting light. This system measures the intensity of emitted light in order to generate a quantitative measure of the protein within the sample. A plate blank was used to adjust for background signal. Values are expressed as signal/μg of total protein, previously determined by BCA assay.

### 2.8 Statistical analysis

**FIGURE 1:**
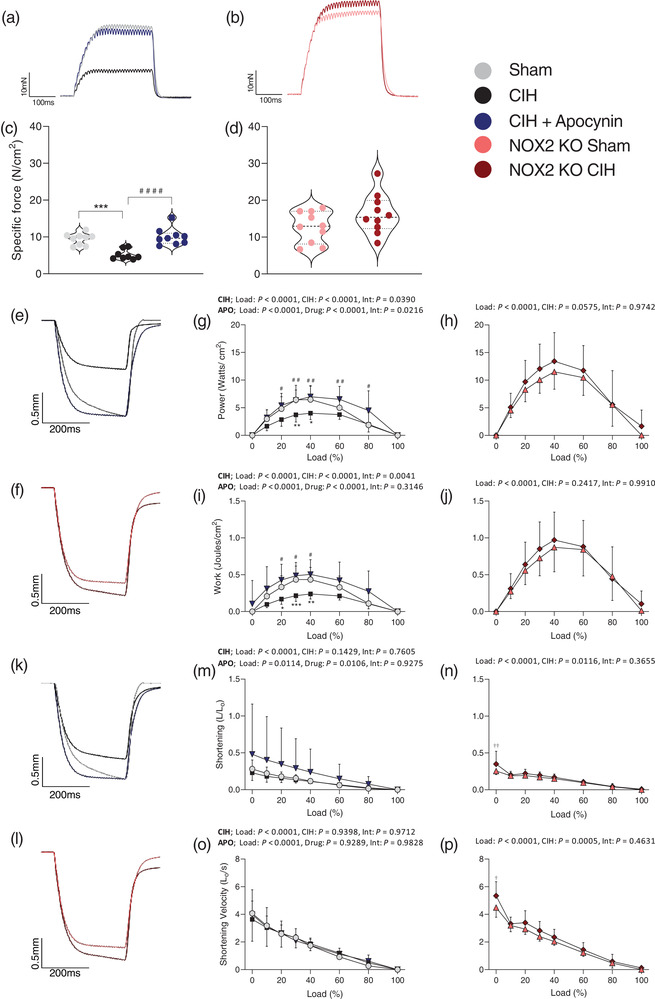
Sternohyoid muscle contractile function *ex vivo*. (a) Original traces of *ex vivo* sternohyoid muscle tetanic contractions for sham (grey), CIH (black) and CIH + APO (blue) preparations. (b) Original traces of *ex vivo* sternohyoid muscle tetanic contractions for NOX2 KO Sham (pink) and NOX2 KO CIH (red) preparations. (c, d) Group data for sternohyoid muscle tetanic force, normalised for muscle CSA, in sham, normoxia (21% O_2_) exposed (*n* = 8); CIH, chronic intermittent hypoxia exposed (*n* = 8); CIH + APO, apocynin (2 mM) given in the drinking water for the duration of CIH exposure (*n* = 9); NOX2 KO, NADPH oxidase 2 knock‐out (B6.129S‐Cybbtm1Din/J) sham (*n* = 8) or CIH‐exposed (*n* = 10) mice. Tetanic force was measured following stimulation at 100 Hz *ex vivo*. Values are expressed as means ± SD. For (c), data sets with confirmed normal distribution were statistically compared using one‐way ANOVA with Tukey's *post hoc* test. Data sets which were not normally distributed were compared using a non‐parametric Kruskal–Wallis test with Dunn's *post hoc* test. Statistical significance was taken at *P* < 0.05. For (d) data sets which were normally distributed were statistically compared using unpaired two‐tailed Student's *t*‐test. Welch's correction was applied in the case of unequal variance. Data that were not normally distributed were compared using the Mann–Whitney non‐parametric test. Statistical significance was taken at *P* < 0.05. (e, k) Original traces of *ex vivo* sternohyoid muscle maximum unloaded shortening for sham (grey), CIH (black) and CIH + APO (blue) preparations. (f, l), original traces of *ex vivo* sternohyoid muscle maximum unloaded shortening for NOX2 KO Sham (pink) and NOX2 KO CIH (red) preparations. For (g, h, i, j, m, n, o, p) data were statistically compared by repeated measures two‐way ANOVA with Bonferroni *post hoc* test. For repeated measures two‐way ANOVA; CIH denotes sham *vs*. CIH; APO denotes CIH versus CIH + APO for (g, i, m, o). Int denotes the interaction between two factors for (g–p). Statistical significance was taken at *P* < 0.05 for Bonferroni *post hoc* test. Relevant comparisons are denoted as follows: sham versus CIH, *P < 0.05, **P < 0.01, ***P < 0.001; CIH versus CIH + APO, ^#^ *P* < 0.05, ^##^ *P* < 0.01, ^####^ *P* < 0.0001; NOX2 KO sham versus NOX2 KO CIH, ^†^ *P* < 0.05, ^††^ *P* < 0.01

Values are expressed as means ± SD. Data were statistically compared using Prism 8.0 (GraphPad Software, San Diego, CA, USA). For the comparison of sham, CIH and CIH + APO groups, data sets with confirmed normal distribution were statistically compared using one‐way ANOVA with Tukey's *post hoc* test. Data sets which were not normally distributed were compared using a non‐parametric Kruskal–Wallis with Dunn's *post hoc* test. Each *P*‐value is adjusted to account for multiple comparisons. Statistical significance was taken at *P* < 0.05. For the comparison of NOX2 KO sham and NOX2 KO CIH groups, data sets which were normally distributed and of equal variance were statistically compared using an unpaired two‐tailed Student's *t*‐test. Welch's correction was applied in the case of unequal variance. Data which were not normally distributed were compared using a Mann–Whitney non‐parametric test. Statistical significance was taken at *P* < 0.05. Isotonic measures of sternohyoid muscle contractile performance (Figure 1) were statistically compared by repeated measures two‐way ANOVA (RMANOVA) with Bonferroni *post hoc* test; *P* < 0.05 was considered statistically significant.

## 3 RESULTS

### 3.1 Sternohyoid muscle contractile function *ex vivo*

Sternohyoid twitch kinetics (twitch force (*P*_t_), CT and ½RT) and isotonic contractile parameters (*P*_max_, *W*_max_, *S*_max_ and *V*_max_) for all groups are shown in Tables 2 and 3. Sternohyoid *P*_t_ was significantly depressed in wild‐type mice following 2 weeks of exposure to CIH compared to wild‐type sham mice (*P* = 0.0076; Table 2); administration of apocynin (2 mM) in the drinking water throughout the exposure to CIH completely prevented this decrease in twitch force (*P* = 0.0028; Table 2). Exposure to CIH had no effect on *P*_t_ in NOX2 KO mice compared to NOX2 KO sham mice (*P* = 0.3063; Table 3). Twitch contraction time (CT) and half‐relaxation time (½RT) were unaffected by exposure to CIH in both wild‐type (Table 2) and NOX2 KO mice (Table 3). Figure 1 shows representative original traces for (Figure 1a) sham (grey), CIH (black), CIH + APO (blue) and (Figure 1b) NOX2 KO sham (pink) and NOX2 KO CIH (red) tetanic contractions. Two weeks of exposure to CIH significantly decreased the force‐generating capacity of the sternohyoid compared to wild‐type sham mice (*P* = 0.0002; Figure 1c). Exposure to CIH resulted in a ∼45% decrease in peak specific force of the sternohyoid compared to sham mice. Apocynin administration throughout the exposure to CIH successfully ameliorated CIH‐induced muscle weakness in wild‐type mice (*P* < 0.0001; Figure 1c). There was no difference in the peak tetanic force produced by NOX2 KO mice exposed to CIH compared to NOX2 KO sham controls (*P* = 0.1147; Figure 1d), revealing a specific role for NOX2 in CIH‐induced sternohyoid muscle weakness. Sternohyoid muscle mass was unaffected by exposure to CIH compared to sham both in wild‐type (*P* = 0.6788; Table 2) and NOX2 KO mice (*P* = 0.4021; Table 3). Co‐treatment with apocynin significantly increased sternohyoid muscle mass compared with CIH exposure alone (*P* = 0.0010; Table 2). Body mass was significantly decreased following exposure to CIH in both wild‐type (*P* = 0.0032; Table 2) and NOX2 KO mice (*P* = 0.0042; Table 3). Co‐treatment with apocynin throughout the CIH exposure prevented the CIH‐induced decrease in mouse body mass (*P* = 0.0047; Table 2).

**TABLE 2:** *Ex vivo* sternohyoid muscle contractile parameters

				*P*		
	**Sham (*n* = 9)**	**CIH (*n* = 9)**	**CIH + APO (*n* = 9)**	**One‐way ANOVA**	**Sham *vs*. CIH**	**CIH *vs*. CIH + APO**
*P*_t_ (N/cm^2^)	1.61 ± 0.39	1.12 ± 0.26	1.67 ± 0.26	**0.0017**	**0.0076**	**0.0028**
CT (ms)	9.83 ± 1.03	10.17 ± 2.83	10.56 ± 1.72	0.2933	—	—
½RT (ms)	1.47 ± 0.22	1.43 ± 0.10	1.56 ± 0.10	0.4147	—	—
*W*_max_ (J/cm^2^)	0.44 ± 0.17	0.25 ± 0.06	0.64 ± 0.24	**0.0006**	0.0985	**0.0004**
*P*_max_ (W/cm^2^)	6.56 ± 2.61	4.36 ± 0.98	7.64 ± 2.28	**0.0128**	0.1120	**0.0103**
*S*_max_ (*L*/*L*_o_)	0.28 ± 0.12	0.23 ± 0.10	0.27 ± 0.05	0.6268	—	—
*V*_max_ (*L*_o_/s)	4.07 ± 1.71	3.64 ± 1.59	4.01 ± 0.97	0.5409	—	—
Muscle mass (mg)	1.66 ± 0.42	1.54 ± 0.14	2.12 ± 0.20	**0.0007**	0.6788	**0.0010**
Body mass (g)	23.6 ± 1.5	21.4 ± 0.7	23.5 ± 1.6	**0.0014**	**0.0032**	**0.0047**

Values are expressed as means ± SD. Data sets with confirmed normal distribution were statistically compared using one‐way ANOVA with Tukey's *post hoc* test. Data sets which were not normally distributed were compared using a non‐parametric Kruskal‐Wallis with Dunn's *post hoc* test. Each *P*‐value is adjusted to account for multiple comparisons. Bolded numbers indicate statistical significance (*P *< 0.05). Abbreviations: CIH, chronic intermittent hypoxia‐exposed; CIH + APO, Apocynin (2 mM) given in the drinking water for the duration of CIH exposure; CT, contraction time; *L*_o_, optimum length; sham, normoxia (21% O_2_)‐exposed; *P*_t_, isometric twitch force; *P*_max_, maximum mechanical power; *S*_max_, maximum shortening; ½RT, half‐relaxation time; *V*_max_, maximum shortening velocity; *W*_max_, maximum mechanical work.

**TABLE 3:** *Ex vivo* sternohyoid muscle contractile parameters

	NOX2 KO sham (*n* = 8)	NOX2 KO CIH (*n* = 10)	NOX2 KO Sham vs. NOX2 KO CIH, *P*
*P*_t_ (N/cm^2^)	2.53 ± 0.46	2.81 ± 0.62	0.3063
CT (ms)	10.94 ± 1.32	11.50 ± 0.85	0.2891
½RT (ms)	1.52 ± 0.21	1.60 ± 0.24	0.1371
*W*_max_ (J/cm^2^)	0.93 ± 0.33	1.03 ± 0.38	0.5877
*P*_max_ (W/cm^2^)	11.98 ± 3.01	14.07 ± 5.01	0.3149
*S*_max_ (*L*/*L*_o_)	0.25 ± 0.05	0.30 ± 0.06	0.0897
*V*_max_ (*L*_o_/s)	4.49 ± 0.70	5.35 ± 1.02	0.0598
Muscle mass (mg)	2.14 ± 0.53	2.39 ± 0.55	0.4021
Body mass (g)	28.1 ± 2.5	25.4 ± 0.9	**0.0042**

Values are expressed as means ± SD. Data sets which were normally distributed were statistically compared using unpaired two‐tailed Student's *t*‐test. Welch's correction was applied in the case of unequal variance. Data which were not normally distributed were compared using a Mann–Whitney non‐parametric test. Statistical significance was taken at *P* < 0.05. Abbreviations: CIH, chronic intermittent hypoxia‐exposed; CT, contraction time; ½RT, half‐relaxation time; *L*_o_, optimum length; NOX2 KO, NADPH oxidase 2 knock‐out (B6.129S‐Cybbtm1Din/J); *P*_t_, isometric twitch force; sham, normoxia‐exposed (21% O_2_); *P*_max_, maximum mechanical power; *S*_max_, maximum shortening; *V*_max_, maximum shortening velocity; *W*_max_, maximum mechanical work.

Figure 1 shows representative original traces of *ex vivo* sternohyoid muscle maximum unloaded shortening for (Figure 1e) sham (grey), CIH (black), CIH + APO (blue) and (Figure 1f) NOX2 KO sham (pink) and NOX2 KO CIH (red) preparations. Figure 1g,h shows the sternohyoid power‐load relationship across all groups. Exposure to CIH significantly decreased the power‐generating capacity of the sternohyoid muscle over a range of loads compared to wild‐type sham mice (*P* < 0.0001; Figure 1g). *Post hoc* analysis revealed a statistically significant decrease in the power‐generating capacity of the sternohyoid following exposure to CIH at 30% (*P *< 0.05) and 40% (*P* < 0.01) of its peak force‐generating capacity when compared to wild‐type sham mice (Figure 1g). The administration of apocynin throughout the exposure to CIH prevented the CIH‐induced decrease in the power‐generating capacity of the sternohyoid over a range of loads compared to mice exposed to CIH alone (*P* < 0.0001; Figure 1g). *Post hoc* analysis revealed a statistically significant increase in the power‐generating capacity of the sternohyoid following treatment with apocynin throughout exposure to CIH at 20%–80% of the peak force‐generating capacity compared to CIH‐exposed mice (Figure 1g). Exposure to CIH had no significant effect on peak power (*P*_max_) compared to wild‐type sham mice (*P* = 0.1120; Table 2); apocynin treatment throughout the exposure to CIH significantly increased *P*_max_ compared to CIH exposure alone (*P* = 0.0103; Table 2). Exposure to CIH had no effect on the power‐generating capacity of the sternohyoid in NOX2 KO mice over the range of loads examined when compared to NOX2 KO sham controls (*P* = 0.0575; Figure 1h). Similarly, *P*_max_ was unaffected by exposure to CIH in NOX2 KO mice (*P* = 0.3149; Table 3). Figure 1i,j, show the sternohyoid work–load relationship across all groups. Exposure to CIH significantly decreased the work produced by the sternohyoid muscle over a range of loads compared to wild‐type sham mice (*P* < 0.0001; Figure 1i). *Post hoc* analysis revealed a statistically significant decrease in mechanical work of the sternohyoid following exposure to CIH at 20% (*P* < 0.05), 30% (*P* < 0.001) and 40% (*P* < 0.01) of the peak force‐generating capacity compared to wild‐type sham mice (Figure 1i). The administration of apocynin throughout the exposure to CIH prevented the CIH‐induced decrease in the work produced by the sternohyoid over a range of loads compared to mice exposed to CIH alone (*P* < 0.0001; Figure 1i). *Post hoc* analysis revealed a statistically significant increase in work produced by the sternohyoid following treatment with apocynin throughout CIH exposure at 20% (*P* < 0.05), 30% (*P* < 0.05) and 40% (*P* < 0.05) of the peak force‐generating capacity compared to CIH‐exposed mice (Figure 1i). Peak work (*W*_max_) was reduced following 2 weeks of exposure to CIH compared to sham mice, but this did not meet the threshold for statistical significance (*P* = 0.0985; Table 2); apocynin administration completely prevented the CIH‐induced decrease in *W*_max_ (*P* = 0.0004; Table 2). Exposure to CIH had no effect on the work produced by the sternohyoid in NOX2 KO mice over the range of loads examined compared to NOX2 KO sham controls (*P* = 0.2417; Figure 1j). Similarly, *W*_max_ was unaffected by exposure to CIH in NOX2 KO mice (*P* = 0.5877; Table 3).

Figure 1 shows representative original traces of *ex vivo* sternohyoid muscle maximum unloaded shortening for (Figure 1k) sham (grey), CIH (black), CIH + APO (blue) and (Figure 1l) NOX2 KO sham (pink) and NOX2 KO CIH (red) preparations. Figure 1m,n shows the sternohyoid shortening–load relationship across all groups. Two weeks of exposure to CIH had no effect on the distance of shortening of the sternohyoid muscle when compared to wild‐type sham mice (*P *= 0.1429; Figure 1m); apocynin treatment throughout the exposure to CIH significantly increased the distance of shortening of the sternohyoid over a range of loads examined compared with exposure to CIH alone (*P* = 0.0106; Figure 1m). Peak shortening (*S*_max_) was unaffected by exposure to CIH or apocynin treatment throughout the CIH exposure in wild‐type mice (*P* = 0.6268; Table 2). NOX2 KO mice exposed to CIH had a significantly increased distance of shortening over the range of loads examined compared to NOX2 KO sham controls (*P* = 0.0116; Figure 1n). *Post hoc* analysis revealed that NOX2 KO CIH‐exposed mice had a significantly increased distance of shortening at 0% of the peak force‐generating capacity compared to NOX2 KO sham mice (*P* < 0.01; Figure 1n). *S*_max_ was unchanged following exposure to CIH in NOX2 KO mice (*P* = 0.0897; Table 3). Figure 1o,p shows the sternohyoid shortening velocity–load relationship across all groups. Two weeks of exposure to CIH had no effect on the shortening velocity of the sternohyoid muscle compared to wild‐type sham mice (*P *= 0.9398; Figure 1o); apocynin treatment throughout the exposure to CIH also had no effect on the shortening velocity of the sternohyoid over a range of loads examined compared with exposure to CIH alone (*P* = 0.9289; Figure 1o). The peak shortening velocity (*V*_max_) was unaffected by exposure to CIH or treatment with apocynin throughout the CIH exposure in wild‐type mice (*P* = 0.5409; Table 2). NOX2 KO mice exposed to CIH had a significantly increased shortening velocity over the range of loads examined compared to NOX2 KO sham controls (*P* = 0.0005; Figure 1p). *Post hoc* analysis revealed that NOX2 KO CIH mice had a significantly increased velocity of shortening at 0% of the peak force‐generating capacity when compared to NOX2 KO sham mice (*P* < 0.05; Figure 1p). *V*_max_ was unaffected by exposure to CIH in NOX2 KO mice (*P *= 0.0598; Table 3).

### 3.2 NOX mRNA and protein expression in sternohyoid muscle

**FIGURE 2:**
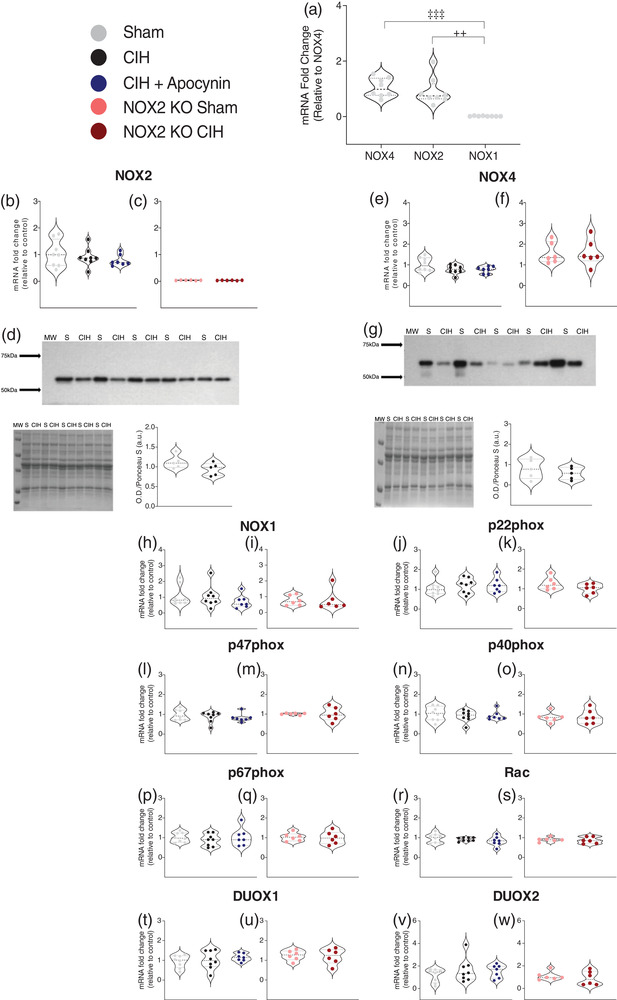
NOX mRNA and protein expression in sternohyoid muscle. (a) Group data (*n* = 6–8 per group) expressed as a fold difference in messenger RNA (mRNA) expression (relative to NOX4) for naïve wild‐type mouse sternohyoid muscle. (b, c), group data (*n* = 6–8 per group) expressed as a fold change in messenger RNA (mRNA) expression (relative to the control group) for sham, CIH, CIH + APO, NOX2 KO sham and NOX2 KO CIH groups. (d) Western Blot of NOX2 in 15 μg of protein extracted from the sternohyoid of mice exposed to 2 weeks of normoxia (sham) or CIH, corresponding Ponceau S‐stained membrane and group data (*n* = 5 per group) for normalised NOX2 expression expressed as arbitrary units (a.u.). (e, f) Group data (*n* = 6–8 per group) expressed as a fold change in mRNA expression (relative to the control group) for sham, CIH, CIH + APO, NOX2 KO sham and NOX2 KO CIH groups. (g) Western Blot of NOX4 in 15 μg of protein extracted from the sternohyoid of mice exposed to 2 weeks of normoxia (sham) or CIH, corresponding Ponceau S‐stained membrane and group data (*n* = 5 per group) for normalised NOX4 expression expressed as arbitrary units (a.u.). (h–w) Group data (*n* = 6–8 per group) expressed as a fold change in mRNA expression (relative to the control group) for sham, CIH, CIH + APO, NOX2 KO sham and NOX2 KO CIH groups for (h, i) NOX1; (j, k) p22phox; (l, m) p47phox; (n, o) p40phox; (p, q) p67phox; (r, s) Rac; (t, u) DUOX1; and (v, w) DUOX2. Values are expressed as mean ± SD. For (a, b, e, h, j, l, n, p, r, t, v), data sets with confirmed normal distribution were statistically compared using one‐way ANOVA with Tukey's *post hoc* test. Data sets which were not normally distributed were compared using a non‐parametric Kruskal‐Wallis test with Dunn's *post hoc* test. Each *P*‐value is adjusted to account for multiple comparisons. Statistical significance was taken at *P* < 0.05. For (c, f, d, g, i, k, m, o, q, s, u, w), data sets which were normally distributed were statistically compared using unpaired two‐tailed Student's *t*‐test. Welch's correction was applied in the case of unequal variance. Data which were not normally distributed were compared using the Mann–Whitney non‐parametric test. Statistical significance was taken at *P* < 0.05. NOX4 versus NOX1: ^‡‡‡^ *P *< 0.001; NOX2 versus NOX1: ^++^ *P *< 0.01. MW, molecular weight marker; NOX1, NADPH oxidase 1; NOX2, NADPH oxidase 2; NOX4, NADPH oxidase 4; NOX2 KO, NADPH oxidase 2 knock‐out (B6.129S‐Cybbtm1Din/J); CIH, chronic intermittent hypoxia‐exposed; CIH + APO, apocynin (2 mM) given in the drinking water for the duration of CIH exposure; sham, normoxia (21% O_2_)‐exposed

Figure 2a shows the mRNA expression of the most predominant muscle‐specific NOX isoforms in naïve wild‐type mouse sternohyoid muscle. The mRNA expression of NOX1 in the sternohyoid was significantly lower than that of both NOX4 (*P* = 0.0002; Figure 2a) and NOX2 (*P* = 0.0002; Figure 2a). There was no difference in the mRNA expression of NOX4 compared with NOX2 (*P* = 0.5346; Figure 2a). mRNA and protein expression of the NOX2 enzyme in mouse sternohyoid muscle is shown in Figure 2b–d. There was no difference in the mRNA expression of NOX2 following exposure to CIH compared with sham in wild‐type mice; apocynin treatment also had no effect on NOX2 mRNA levels (Figure 2b). An ablation of NOX2 mRNA was demonstrated in NOX2 KO sternohyoid muscle (Figure 2c). Western blot bands were detected at approximately 65 kDa, corresponding to the predicted molecular mass of the NOX2 subunit (Figure 2d). Figure 2d also shows the corresponding Ponceau S‐stained membrane, confirming relatively equal protein loading and electrotransfer. Densitometric analysis of NOX2 band intensities, normalised by the intensity of the corresponding Ponceau S‐stained proteins, revealed no significant difference in the protein expression of the NOX2 subunit in the sternohyoid of mice exposed to CIH compared with sham in wild‐type mice (Figure 2d; *P* = 0.120). mRNA and protein expression of the NOX4 enzyme in mouse sternohyoid muscle is shown in Figure 2e–g. There was no difference in the mRNA expression of NOX4 following exposure to CIH compared with sham in wild‐type mice; apocynin treatment also had no effect on NOX4 mRNA levels (Figure 2e). Exposure to CIH also had no effect on NOX4 mRNA expression in NOX2 KO mice (Figure 2e). Western blot bands were detected at approximately 63 kDa, corresponding to the predicted molecular mass of the NOX4 subunit (Figure 2e). Figure 2e also shows the corresponding Ponceau S‐stained membrane, confirming relatively equal protein loading and electro‐transfer. Densitometric analysis of NOX4 band intensities, normalised by the intensity of the corresponding Ponceau S‐stained proteins, revealed no difference in the protein expression of the NOX4 subunit in the sternohyoid of mice exposed to CIH compared to sham in wild‐type mice (Figure 2g; *P* = 0.419). The mRNA expression of NOX catalytic and accessory subunits in sternohyoid muscle is shown in Figure 2h–w. There was no statistically significant difference in the mRNA expression of NOX1 (Figure 2h, i), p22phox (Figure 2j, k), p47phox (Figure 2l, m), p40phox (Figure 2n, o), p67phox (Figure 2p, q), Rac (Figure 2r, s), DUOX1 (Figure 2t, u), or DUOX2 (Figure 2v, w) across all groups. Two weeks of exposure to CIH caused a significant increase in NOX enzymatic activity in sternohyoid muscle compared with sham in wild‐type mice (Table 4; *P* = 0.0009).

**TABLE 4:** Indices of redox balance, protein synthesis and degradation in sternohyoid homogenates from sham and CIH exposed mice

	Sham (*n* = 8)	CIH (*n* = 8)	Sham *vs*. CIH, *P*
NADPH oxidase activity (nmol/min/μg)	0.55 ± 0.13	0.79 ± 0.04	**0.0009**
TBARS (nM/mg)	19.18 ± 3.42	25.49 ± 8.98	0.0962
Citrate synthase activity (μmole/mg)	0.28 ± 0.04	0.25 ± 0.06	0.1815
HIF‐1α (signal/μg)	1.88 ± 0.58	1.46 ± 0.39	0.1146
Phospho‐FOXO‐3a (signal/μg)	51.32 ± 12.01	48.84 ± 7.47	0.6279
Phopho‐ERK1/2 (signal/μg)	18.43 ± 5.67	22.25 ± 7.46	0.2677
Phospho‐JNK (signal/μg)	82.45 ± 28.59	110.11 ± 78.53	0.7984
Phospho‐p38 (signal/μg)	9.88 ± 1.11	9.65 ± 1.52	0.7288

Values are expressed as mean ± SD. Data sets which were normally distributed were statistically compared using unpaired two‐tailed Student's *t* test. Welch's correction was applied in the case of unequal variance. Data which were not normally distributed were compared using Mann Whitney non‐parametric tests. Statistical significance was taken at *P* < 0.05. Abbreviations: CIH, chronic intermittent hypoxia‐exposed; ERK 1/2, extracellular‐signal‐regulated kinase 1/2; FOXO‐3a, forkhead box O3a; HIF‐1α, hypoxia‐inducible factor 1‐α; JNK, c‐Jun N‐terminal kinase; Sham, normoxia (21% O_2_)‐exposed; TBARS, thiobarbituric acid reactive substances.

### 3.3 Indices of redox balance and protein synthesis and degradation in sternohyoid homogenates from sham and CIH‐exposed mice

Various indices of redox balance, protein synthesis and degradation in the sternohyoid of sham and CIH‐exposed wild‐type mice are shown in Table 4. Two weeks of exposure to CIH had no effect on levels of TBARS (a marker of lipid peroxidation) in the sternohyoid compared to sham controls (*P* = 0.0962; Table 4). Similarly, exposure to CIH had no effect on citrate synthase activity (*P* = 0.1825; Table 4; indicative of mitochondrial integrity) or HIF‐1α (*P *= 0.1146; Table 4; master regulator of the hypoxic response) protein content compared to sham. Exposure to CIH had no effect on the expression of key proteins involved in protein synthesis and degradation (phospho‐FOXO‐3α, phopho‐ERK1/2, phospho‐JNK and phospho‐p38) in skeletal muscle when compared to sham controls.

### 3.4 mRNA expression of genes relating to myogenesis in sternohyoid muscle

**FIGURE 3:**
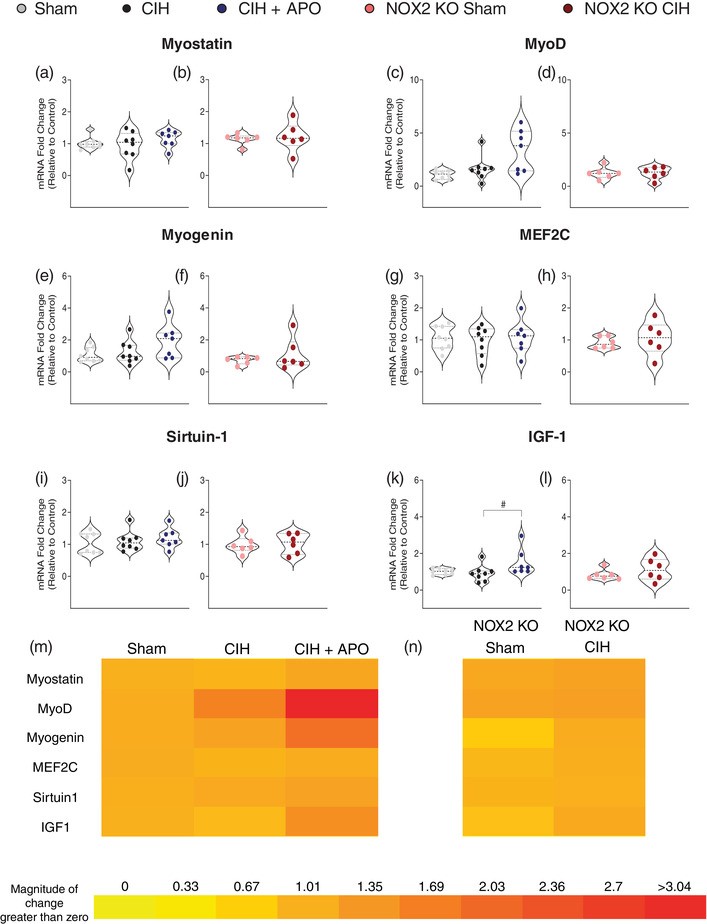
mRNA expression of genes relating to myogenesis in sternohyoid muscle. (a–l) Group data (*n* = 6–8 per group) expressed as a fold change in mRNA expression (relative to the control (sham) group) for sham, CIH, CIH + APO, NOX2 KO sham and NOX2 KO CIH groups for (a, b) myostatin; (c, d) MyoD; (e, f) myogenin; (g, h) MEF2C; (i, j) sirtuin‐1; and (k, l) IGF‐1. (m, n) Heat map depicting the fold changes in mRNA expression for (m) Sham, CIH and CIH + APO and (n) NOX2 KO Sham and NOX2 KO CIH groups. Red represents an increase and yellow represents a decrease in expression. Values are expressed as means ± SD. For (a, c, e, g, i, k), data sets with confirmed normal distribution were statistically compared using one‐way ANOVA with Tukey's *post hoc* test. Data sets which were not normally distributed were compared using a non‐parametric Kruskal–Wallis test with Dunn's *post hoc* test. Each *P*‐value is adjusted to account for multiple comparisons. Statistical significance was taken at *P* < 0.05. For (b, d, f, h, j, l), data sets which were normally distributed were statistically compared using unpaired two‐tailed Student's *t*‐test. Welch's correction was applied in the case of unequal variance. Data which were not normally distributed were compared using the Mann–Whitney non‐parametric test. Statistical significance was taken at *P* < 0.05. Relevant comparisons are denoted as follows: CIH versus CIH + APO: ^#^ *P* < 0.05. IGF‐1, insulin‐like growth factor 1; MEF2C, myocyte‐specific enhancer factor 2C; MyoD, muscle differentiation protein 1

Figure 3 shows the mRNA expression of genes relating to the muscle differentiation process in sternohyoid muscle across all groups. There were no statistically significant changes in the mRNA expression of myostatin (Figure 3a,b), muscle differentiation protein 1 (MyoD; Figure 3c,d), myogenin (Figure 3e,f), myocyte‐specific enhancer factor 2C (MEF2C; Figure 3g,h), sirtuin‐1 (Figure 3i,j) or insulin‐like growth factor 1 (IGF‐1; Figure 3k,l) across all groups. Figure 3m,n shows heat maps summarising the sternohyoid mRNA expression data for these genes across all groups examined.

### 3.5 mRNA expression of genes relating to antioxidant status in sternohyoid muscle

**FIGURE 4:**
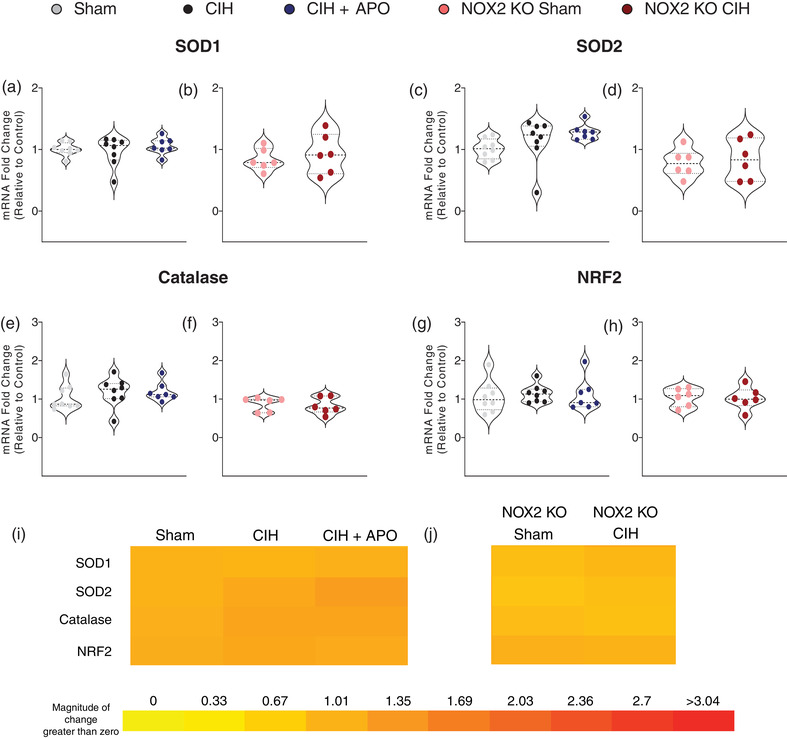
mRNA expression of genes relating to antioxidant status in sternohyoid muscle. (a–h) Group data (*n* = 6–8 per group) expressed as a fold change in mRNA expression (relative to the control (sham) group) for sham, CIH, CIH + APO, NOX2 KO sham and NOX2 KO CIH groups for (a, b) SOD1; (c, d) SOD2; (e, f) catalase; and (g, h) NRF2. (i, j) Heat map depicting the fold changes in mRNA expression for (i) Sham, CIH, CIH + APO and (j) NOX2 KO Sham and NOX2 KO CIH groups. Red represents an increase and yellow represents a decrease in expression. For (a, c, e, g), data sets with confirmed normal distribution were statistically compared using one‐way ANOVA with Tukey's *post hoc* test. Data sets which were not normally distributed were compared using a non‐parametric Kruskal–Wallis test with Dunn's *post hoc* test. Each *P*‐value is adjusted to account for multiple comparisons. Statistical significance was taken at *P* < 0.05. For (b, d, f, h), data sets which were normally distributed were statistically compared using unpaired two‐tailed Student's *t‐*test. Welch's correction was applied in the case of unequal variance. Data which were not normally distributed were compared using the Mann–Whitney non‐parametric test. Statistical significance was taken at *P* < 0.05. NRF2, nuclear factor erythroid 2‐related factor 2; SOD1, superoxide dismutase 1; SOD2, superoxide dismutase 2

Figure 4 shows the mRNA expression of genes relating to antioxidant capacity in sternohyoid muscle across all groups. There were no statistically significant changes in the mRNA expression of superoxide dismutase (SOD) 1 (Figure 4a,b), SOD2 (Figure 4c,d), catalase (Figure 4e,f) or myocyte‐specific enhancer factor 2C (NRF2; Figure 4g,h), across all groups. Figure 4i,j, shows heat maps summarising the sternohyoid mRNA expression data for these genes across all groups examined.

### 3.6 mRNA expression of genes relating to inflammation and protein degradation in sternohyoid muscle

**FIGURE 5:**
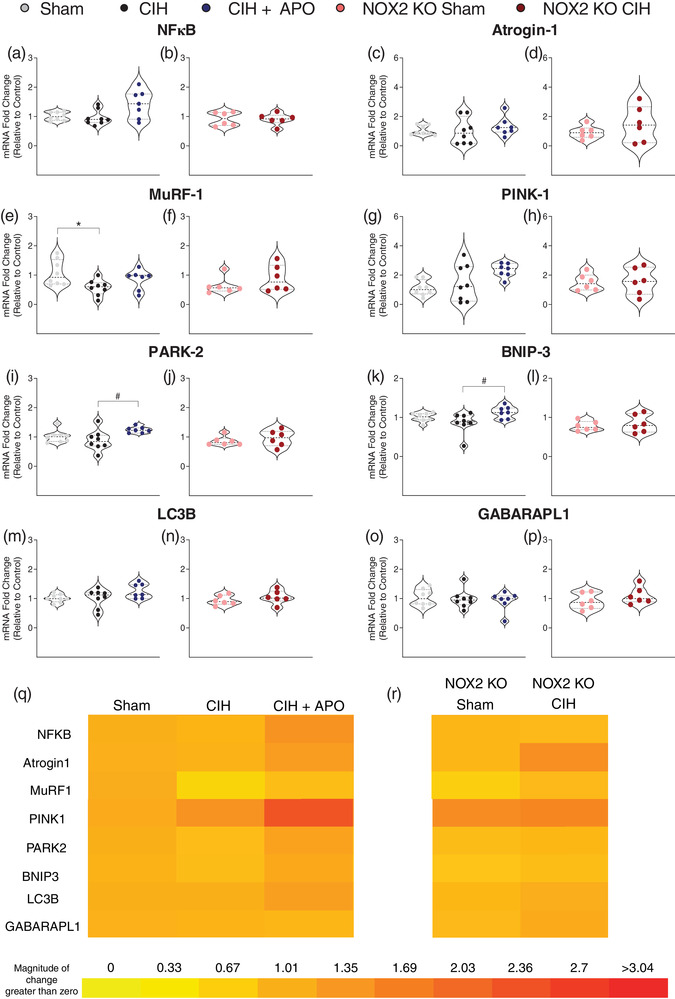
mRNA expression of genes relating to inflammation and protein degradation processes in sternohyoid muscle. (a–p) Group data (*n* = 6–8 per group) expressed as a fold change in mRNA expression (relative to the control (sham) group) for sham, CIH, CIH + APO, NOX2 KO sham and NOX2 KO CIH groups for (a, b) NF‐κB; (c, d) atrogin‐1; (e, f) MuRF1; (g, h) PINK1; (i, j) PARK2; (k, l) BNIP3; (m, n) LC3B; (o, p) GAPABARPL1. (q, r) Heat map depicting the fold changes in mRNA expression for (q) Sham, CIH, CIH + APO and (r) NOX2 KO Sham and NOX2 KO CIH groups. Red represents an increase and yellow represents a decrease in expression. For (a, c, e, g, i, k, m, o), data sets with confirmed normal distribution were statistically compared using one‐way ANOVA with Tukey's *post hoc* test. Data sets which were not normally distributed were compared using a non‐parametric Kruskal–Wallis test with Dunn's *post hoc* test. Each *P*‐value is adjusted to account for multiple comparisons. Statistical significance was taken at *P* < 0.05. For (b, d, f, h, j, l, n, p), data sets which were normally distributed were statistically compared using unpaired two‐tailed Student's *t*‐test. Welch's correction was applied in the case of unequal variance. Data which were not normally distributed were compared using the Mann–Whitney non‐parametric test. Statistical significance was taken at *P* < 0.05. Relevant comparisons are denoted as follows: sham vs. CIH: *P < 0.05; CIH versus CIH + APO: ^#^ *P* < 0.05. BNIP3, bcl‐2 19 kDa interacting protein 3; GABARAPL1, γ‐aminobutyric acid receptor‐associated protein‐like 1; LC3B, microtubule‐associated proteins 1A/1B light chain 3B; MuRF1, muscle RING finger 1; NF‐κB, nuclear factor κ‐light‐chain‐enhancer of activated B cells; PARK2, parkin; PINK1, PTEN‐induced kinase 1

Figure 5 shows the mRNA expression of genes relating to inflammation and protein degradation in sternohyoid muscle across all groups. Exposure to 2 weeks of CIH significantly decreased the mRNA expression of muscle RING finger 1 (MuRF‐1) compared to wild‐type sham mice (*P* = 0.0448; Figure 5e); the administration of apocynin throughout the CIH exposure did not ameliorate this decrease (*P* = 0.4260; Figure 5e). Exposure to CIH had no effect on the mRNA expression of MuRF‐1 in NOX2 KO mice (*P* = 0.4848; Figure 5f). Exposure to CIH for 2 weeks had no effect on the mRNA expression of parkin (PARK‐2) in the sternohyoid compared to wild‐type sham mice (*P* = 0.5448; Figure 5i); however, the administration of apocynin throughout the exposure to CIH significantly increased the mRNA expression of PARK‐2 compared with CIH exposure alone (*P *= 0.0275; Figure 5i). Exposure to CIH had no effect on the mRNA expression of PARK‐2 in NOX2 KO mice (*P* = 0.4979; Figure 5j). CIH exposure had no effect on the mRNA expression of bcl‐2 19 kDa interacting protein 3 (BNIP‐3) in the sternohyoid compared to wild‐type sham mice (*P* = 0.6921; Figure 5k); apocynin treatment throughout the exposure to CIH significantly increased the mRNA expression of BNIP‐3 (*P* = 0.0237; Figure 5k). Exposure to CIH had no effect on the mRNA expression of BNIP‐3 in NOX2 KO mice (*P* = 0.5814; Figure 5l). There were no statistically significant changes in the mRNA expression of nuclear factor κ‐light‐chain‐enhancer of activated B cells (NF‐κB; Figure 5a,b), atrogin‐1 (Figure 5c,d), PTEN‐induced kinase 1 (PINK‐1; Figure 5g,h), microtubule‐associated proteins 1A/1B light chain 3B (LC3B; Figure 5m,n) or γ‐aminobutyric acid receptor‐associated protein‐like 1 (GABARAPL1; Figure 5o,p) across all groups. Figure 5q,r, shows heat maps summarising the sternohyoid mRNA expression data for these genes across all groups examined.

## 4 DISCUSSION

The structure and function of upper airway muscles are altered in patients with OSAS, with functional deficits contributing to upper airway collapse (Carrera et al., 1999; Sériès et al., 1996). While the mechanisms underpinning aberrant upper airway muscle remodelling in OSAS are likely multifactorial, a wealth of research has implicated exposure to CIH as a major factor. CIH‐induced upper airway muscle weakness and/or fatigue has been described in animal models utilising a variety of intermittent hypoxia paradigms (Ding & Liu, 2011; Dunleavy et al., 2008; Jia & Liu, 2010; Liu et al., 2005, Liu et al., 2009; McDonald et al., 2015; Pae et al., 2005; Skelly et al., 2012b; Wang et al., 2013; Zhou & Liu, 2013). Similarly, 2 weeks of exposure to CIH resulted in significant sternohyoid muscle weakness in our model, evidenced by a ∼45% reduction in peak force‐generating capacity.

Antioxidant supplementation has previously been shown to ameliorate or prevent upper airway muscle dysfunction following exposure to CIH in rat models (Dunleavy et al., 2008; Liu et al., 2005; Skelly et al., 2012b; Skelly et al., 2013). NOX enzymes have been proposed as a source of ROS contributing to skeletal muscle dysfunction in a range of models (Ahn et al., 2015; Regan et al., 2017; Whitehead et al., 2010; Williams et al., 2015). In the current study, the administration of apocynin (a NOX2 inhibitor) throughout the exposure to CIH entirely prevented sternohyoid muscle weakness, restoring muscle force to levels exceeding sham animals. This extends previous work (Dunleavy et al., 2008; Liu et al., 2005; Skelly et al., 2012b, 2013) and implicates NOX2 as the specific source of ROS underpinning CIH‐induced muscle weakness in our model. However, while commonly used as a NOX2 inhibitor, apocynin is also reported to function as a general antioxidant (Altenhöfer et al., 2015). To account for this and ensure the specificity of our findings, transgenic NOX2 KO mice were employed. We demonstrated that CIH‐induced muscle weakness is absent in NOX2 KO mice, as evidenced by an absence of deficits in the peak force‐generating capacity, work or power produced by the sternohyoid following exposure to CIH in mice lacking the NOX2 enzyme. This confirms that NOX2‐derived ROS are entirely responsible and necessary for CIH‐induced sternohyoid muscle weakness.

CIH‐induced increases in NOX enzyme expression and or/activity have been reported in a wide range of organs and tissues. This highlights a role for NOX‐dependent ROS in CIH‐induced alterations to homeostatic control. The regulation of NOX enzymes and their associated subunits is highly complex with evidence for the transient and sequential activation of NOX, such that understanding the patterns of NOX expression, activity and isoform cross‐talk is a challenging task (Brandes et al., 2014). Two weeks of exposure to CIH has been shown to increase the protein expression of NOX2, and its activator subunit, p47phox, in rat sternohyoid muscle (Williams et al., 2015), suggestive of a role for NOX2‐dependent ROS in CIH‐induced upper airway muscle weakness (Skelly et al., 2012b). In contrast to this, we observed no alteration to the mRNA or protein expression of NOX2 or the mRNA expression of p47phox, p22phox, p67phox, p40phox or Rac in the mouse sternohyoid muscle in the current study. While transcriptional regulation of NOX2 has been observed, NOX2 is thought to be predominantly acutely regulated by post‐translational mechanisms such as the phosphorylation of regulatory cytosolic subunits, thus enabling the enzyme to assemble as a fully functioning complex (Lambeth, 2004). Of interest, apocynin had no effect on NOX2 mRNA expression in the sternohyoid. Apocynin inhibits the generation of superoxide by NOX by blocking migration of p47phox to the membrane, thus interfering with assembly of the functional NOX complex (Touyz, 2008). Therefore, the lack of an effect of apocynin on NOX2 mRNA expression in the current study may be explained by the fact that apocynin acutely inhibits NOX activity, rather than its transcriptional expression.

We reason that the increase in NOX activity in the sternohyoid following exposure to CIH in our model is NOX2‐dependent given that NOX2 is typically acutely regulated and NOX2 KO prevents CIH‐induced muscle weakness in our model. However, we acknowledge that our results must be interpreted with caution as the NOX activity assay utilised in the current study is not specific for any one isoform of NOX; rather it functions to assess the rate of NADPH consumption. NAD(P)H consumption assays have previously been used in the assessment of NOX activity in skeletal muscle (Adams et al., 2005; Ahn et al., 2015; Bowen, Mangner et al., 2015; Bowen, Rolim et al., 2015; Javeshghani et al., 2002). However, a recent study shows that these assays are not specific to NOX activity and, in several tissues and cell types, the signal generated was unchanged with a triple NOX1, NOX2 and NOX4 knockout (Rezende et al., 2016). Indeed, the lack of specific NOX activity measurements has been a major impediment in the field, and this warrants further attention. Moreover, we did not assess NOX activity in CIH+APO or NOX2 null tissue, which is a limitation of the study as we have not provided validation of the specificity of the NADPH consumption assay. We acknowledge also that NOX2‐dependent ROS may have been produced by macrophage recruited to hypoxic tissue, but this was not assessed in our study.

There is evidence of increased oxidative stress in a variety of tissues across animal models of CIH, consistent with the observation that human OSAS is an oxidative stress disorder (Lavie, 2003). However, despite the clear link between NOX2‐dependent ROS and CIH‐induced UA muscle weakness in our model, we report no evidence of concomitant overt oxidative stress in the sternohyoid following exposure to CIH. In the present study, we report no alteration to the mRNA expression of the master cellular regulator of antioxidant response, NRF2, or the endogenous antioxidants, SOD1, SOD2 and catalase, in the sternohyoid following exposure to CIH. As a result, we suggest that any putative CIH‐induced challenge to redox homeostasis does not manifest as a depletion of the antioxidant defence system (Ding & Liu, 2011; Williams et al., 2015), nor does it evoke an adaptive response in the form of an increase in antioxidant enzyme expression. However, we acknowledge that our results only reflect changes at the transcriptional level of the antioxidant defence system and that CIH‐induced alterations to antioxidant enzyme activity cannot be ruled out.

Levels of TBARS show a positive correlation to OSAS severity as determined by increased apnoea index (Kent et al., 2011). Five weeks of exposure to CIH has been shown to increase lipid peroxidation, evidenced by increased MDA levels in the genioglossus of female rats (Ding & Liu, 2011). Conversely, 2 weeks of exposure to CIH was insufficient to alter levels of lipid peroxidation, determined by levels of 4‐HNE, in the sternohyoid in a rat model of CIH (Williams et al., 2015). Consistent with the latter study, we report that 2 weeks of exposure to CIH had no effect on levels of TBARS in the mouse sternohyoid, and thus oxidative stress in the form of lipid peroxidation appears to be absent in our model.

Citrate synthase activity is a routinely used marker of mitochondrial integrity and aerobic capacity, as citrate synthase is the initial enzyme of the TCA cycle. Citrate synthase activity was unaffected by exposure to CIH in the mouse sternohyoid in the current study, consistent with unaltered citrate synthase activity previously reported in a rat model of CIH (Williams et al., 2015). Similarly, other metabolic markers, including succinate dehydrogenase (SDH) and glycerol phosphate dehydrogenase (GPDH) activities, have been shown to be unaltered in rat sternohyoid following exposure to CIH (Skelly et al., 2012a, 2012b).

Impaired mitochondria are removed or rejuvenated via autophagy. An increase in key regulators of mitophagy has been observed in rat genioglossus following 2–3 weeks of exposure to CIH, with these markers of mitophagy subsequently decreasing for the remaining 2 weeks of exposure to CIH (Wang et al., 2021). In the current study, we examined the mRNA expression of the PINK1/PARK2 pathway, which has emerged as a principal regulator of mitochondrial degradation in a range of biological systems (Narendra & Youle, 2011). However, we found that exposure to CIH had no effect on the mRNA expression of PINK1 or PARK2 in the sternohyoid. Our data suggest that overt oxidative stress and subsequent mitochondrial dysfunction are not likely to underlie the CIH‐induced alteration to sternohyoid muscle function observed in the current study.

We report no indication of muscle atrophy in the sternohyoid following exposure to CIH in the current study, as evidenced by the unchanged mRNA expression of key muscle‐specific ubiquitin ligases, MuRF1 and atrogin‐1. Similarly, key cell signalling pathways pivotal in maintaining the balance between atrophy and hypertrophy in skeletal muscle, including mitogen‐activated protein kinase and FOXO‐3a, appear unaltered by exposure to CIH. Basal levels of autophagy are necessary for the homeostatic maintenance of muscle mass. However, an interplay between atrophy and autophagy has been demonstrated to underlie a loss of muscle mass and resultant weakness in skeletal muscle (Dobrowolny et al., 2008). Consistent with a lack of evidence for CIH‐induced muscle atrophy in the sternohyoid, we also report unaltered mRNA levels of key autophagy markers, GABARAPL1, LC3B and BNIP3.

Five weeks of exposure to CIH has been shown to decrease the expression of key myogenic regulatory factors (MRFs), MyoD and myogenin, in the genioglossus of rats (Zhou & Liu, 2013). The authors suggest that exposure to CIH damaged genioglossus regenerative properties, which may have implications for the observed decrease in genioglossus fatigue resistance. In the present study, we report no alterations to the mRNA expression of key MRFs including MyoD, myostatin, myogenin, sirtuin‐1 and MEF2C in the sternohyoid following exposure to CIH. These gene expression data, in conjunction with our functional results demonstrating that exposure to CIH does not affect the distance or velocity of shortening in the sternohyoid muscle, suggest that CIH‐induced NOX2‐dependent alterations to UA muscle performance are not likely to be underpinned by structural remodelling.

In view of the collective data described above, our results are consistent with previous views (Williams et al., 2015) that CIH‐induced sternohyoid muscle weakness may be mediated by altered redox signalling, perhaps within microdomains of the muscle, evidently with no widespread cellular stress or structural alterations. Our functional data revealed no change in muscle contractile kinetics, suggesting no major redox modulation of Ca^2+^ handling. This suggests that muscle weakness in CIH‐induced sternohyoid may be an effect of redox modulation at the level of the myofilaments influencing cross‐bridge cycling or Ca^2+^ sensitivity of the contractile apparatus through redox modulation of troponin. Notably, force loss in CIH‐exposed muscle was especially evident at high frequency stimulation (i.e., 100 Hz), suggesting impaired force‐generating capacity of cross‐bridges in CIH muscles, since both reduced SR Ca^2+^ release and decreased myofibrillar Ca^2+^ sensitivity have greater implications for force loss at low stimulus frequencies due to the non‐linear relationship between force and myoplasmic free [Ca^2+^] (Westerblad et al., 2000).

The specific mechanism by which NOX2‐derived ROS underlie CIH‐induced upper airway muscle weakness remains elusive. We observed a ∼30% increase in the peak force‐generating capacity of the sternohyoid in NOX2 KO mice, compared with wild‐type controls. Similarly, we observed a significant increase in the power and work produced by the sternohyoid in NOX2 KO mice across the load continuum examined. Our findings extend previous work (Skelly et al., 2010; Skelly et al., 2012b; Williams et al., 2015) to suggest that NOX2 is an important source of basal ROS production in mouse sternohyoid muscle. Our novel data highlight that NOX2‐derived ROS are powerful inhibitors of sternohyoid force and power, and by extension it is therefore plausible to suggest that increased ROS associated with exposure to CIH directly impairs sternohyoid muscle function.

### 4.1 Conclusion

The significance of our findings is that exposure to CIH, a hallmark feature of human OSAS, is detrimental to the maintenance of upper airway patency, potentially increasing the risk of obstructive airway events throughout the night cycle. Therefore, exposure to CIH could establish a vicious cycle serving to exacerbate respiratory morbidity in human OSAS. Therapeutic strategies to improve upper airway muscle performance may function as an adjunct therapy to reduce OSAS severity in human OSAS. Our data suggest that NOX2 is a viable target for pharmacotherapeutic intervention.

## COMPETING INTERESTS

The authors confirm no conflict of interest.

## AUTHOR CONTRIBUTIONS

Conceptualisation of experimental design: S.D., D.B., V.H., K.O.H. Experimental procedures: S.D., D.B., S.E., O.Z. Data analysis: S.D. Drafting of the original manuscript: S.D., K.O.H. All authors have read and approved the final version of this manuscript and agree to be accountable for all aspects of the work in ensuring that questions related to the accuracy or integrity of any part of the work are appropriately investigated and resolved. All persons designated as authors qualify for authorship, and all those who qualify for authorship are listed.

## Supporting information

Statistical Summary Document

## Data Availability

Raw data were generated at the Department of Physiology, University College Cork. The data supporting the findings of this study are available from the authors on request.
